# Development of an accelerated cellular model for early changes in Alzheimer’s disease

**DOI:** 10.1038/s41598-023-45826-5

**Published:** 2023-10-26

**Authors:** Huijing Xue, Sylvester Gate, Emma Gentry, Wolfgang Losert, Kan Cao

**Affiliations:** 1 Department of Cell Biology and Molecular Genetics, University of Maryland, College Park, MD 20742 USA; 2 Institute of Physical Sciences, University of Maryland, College Park, MD 20742 USA

**Keywords:** Cell biology, Developmental biology, Drug discovery, Neuroscience

## Abstract

Alzheimer’s Disease (AD) is a leading cause of dementia characterized by amyloid plaques and neurofibrillary tangles, and its pathogenesis remains unclear. Current cellular models for AD often require several months to exhibit phenotypic features due to the lack of an aging environment in vitro. Lamin A is a key component of the nuclear lamina. Progerin, a truncated protein resulting from specific lamin A mutations, causes Hutchinson–Gilford Progeria Syndrome (HGPS), a disease that prematurely ages individuals. Studies have reported that lamin A expression is induced in the brains of AD patients, and overlapping cellular phenotypes have been observed between HGPS and AD cells. In this study, we investigated the effects of exogenous progerin expression on neural progenitor cells carrying familial AD mutations (FAD). Within three to four weeks of differentiation, these cells exhibited robust AD phenotypes, including increased tau phosphorylation, amyloid plaque accumulation, and an elevated Aβ42 to Aβ40 ratio. Additionally, progerin expression significantly increased AD cellular phenotypes such as cell death and cell cycle re-entry. Our results suggest that progerin expression could be used to create an accelerated model for AD development and drug screening.

## Introduction

The nuclear lamina, located under the inner nuclear membrane, provides structural support to the nucleus and interacts with chromatin and inner nuclear membrane proteins^1^. It mainly consists of two types, A-type lamins (lamin A and lamin C) and B-type lamins (lamin B1 and lamin B2). Lamin A and lamin C are the alternatively spliced products of the *LMNA* gene^2^, and lamin B1 and lamin B2 are encoded by *LMNB1*^3^ and *LMNB2*^4^*,* respectively. Mutations in *LMNA* lead to a wide range of diverse diseases called laminopathies. In particular, Hutchinson–Gilford Progeria Syndrome (HGPS), a devastating premature aging disease, is mainly caused by a point mutation in exon 11 of the *LMNA* gene, G608G (GGC > GGT), resulting in a cryptic splicing event and generating a truncated product called progerin^5^. The truncated part contains a ZMPSTE24 cleavage site, which is responsible for the cleavage of the farnesylated tail in prelamin A^5^. Thus, progerin is permanently farnesylated and becomes attached to the inner nuclear membrane (INM)^6^. The accumulation of progerin inside the nucleus causes misshaped nuclear morphology, lamin B1 downregulation, chromatin relaxation, and apoptosis^7^, which overlap with the phenotypes in the Alzheimer's Disease (AD) model^8^.

AD is a neurodegenerative disease, and one of the most common causes of dementia^9^. It can be subtyped into two categories. One is familial AD (FAD), which begins before age 65, and the other is sporadic AD (SAD), which usually begins after age 65^10^. Two hallmarks of AD are senile plaques made up of β-amyloid (Aβ), and neurofibrillary tangles (NFTs) made up of tau protein^11^. Both genetic and environmental risk factors can contribute to the formation of plaques and fibrillary tangles, with aging being one of the greatest risk factors^12^. However, the specific mechanism underlying how these protein aggregations form remains unclear. One leading hypothesis is the Aβ cascade hypothesis. Aβ is derived from the Amyloid Precursor Protein (APP), and it has different isoforms, Aβ40, Aβ42, and Aβ43, which are the products of ﻿heterogeneous γ-secretase cleavage^13^. Starting from monomers, Aβ aggregates into oligomers and fibrils, ﻿causing neurotoxicity and leading to neuronal cell death and neurodegeneration^14^. Aβ42 is highly self-aggregating and, therefore, potentially promotes brain Aβ deposits^15,16^; thus, Aβ42 to Aβ40 ratio is considered a sensitive diagnostic marker^17^. Another main hypothesis for an AD mechanism involves tau phosphorylation. Tau belongs to Microtubule-Associated Proteins (MAPs) and acts as a microtubule stabilizer in the axon^18^. Tau protein is hyperphosphorylated in the brains of AD patients, and these abnormally phosphorylated proteins form paired helical filaments (PHF), and further form NFTs^19^. There are different sites of tau phosphorylation, including ﻿Tyr18, Ser199, Ser202/Thr205, Thr231, Ser262, Ser396, and Ser422^20^. Whether Aβ cascade and tau phosphorylation are causal or synergistic is still under investigation^21–23^. Both of these pathways are reported to be involved in AD events, including cell cycle re-entry, cell apoptosis, and oxidative stress^24–26^.

Several studies have suggested the potential involvement of the nuclear lamina in AD. Researchers reported the changes in nuclear morphology in various AD models^27,28^. Despite the low expression level of lamin A in the brain, one group observed that both transcriptional and translational levels of lamin A were significantly increased in the hippocampus in late-stage AD^29^. Another group also observed a significant increase of lamin A expression and reinforced perinuclear Lamin B2 in hippocampal neurons through AD progression^30^. Besides AD, a strong upregulation of lamin A can be detected in the brain of Alexander disease (AxD) patients, and it is associated with increasing brain stiffness^31^. Moreover, overexpression of A-type lamins, including the mutant progerin, is known to negatively impact B-type lamins' expression^32^, which can induce heterochromatin relaxation in AD^28^. These studies suggest that lamin A accumulation may play a role in neurodegeneration.

Currently, most of the AD drug candidates (> 99.6% since 2002) in clinical trials fail to demonstrate sufficient clinical efficacy^33^. A significant challenge in Alzheimer's disease research is the absence of standardized and effective models. There are three main types of experimental models available: animal models, human post-mortem tissues, and cell culture models. Animal models are typically time-consuming and expensive. The majority of AD animal models are transgenic mice, with over 200 models developed to date (according to Alzforum). However, most clinical trials have failed, despite the efficacy of the compounds used to slow the disease in mouse models^34,35^. The reasons for the lack of translational success in mouse models are complicated. Due to the sequence differences between mouse Aβ and human Aβ^36^, most mouse models utilized one or multiple FAD mutations to introduce transgenic human Aβ. Although these FAD models can generate some AD features, none of them can recapitulate the complete disease profile. Most of them do not show either amyloid plaques or tau tangles^37^. Mouse and human tau share only 88% sequence homology and endogenous mouse tau could inhibit the aggregation of human tau^38,39^. This could explain the lack of tau tangles in these transgenic mice. More recently, knock-in mice were developed, which were considered more physiologically relevant^40^. However, the pathological phenotypes and disease progression are still inconsistent, and if these knock-in mouse models are representative still needs to be validated. Because of the lack of high-quality human post-mortem tissues, cell culture models have become a popular choice. Most AD cell culture models are derived from human induced pluripotent stem cells (iPSCs)^41^. However, iPSC-derived neurons can be rejuvenated after reprogramming^42^. This poses a concern since AD is an age-related disease, which could explain why many current iPSC-derived models are time-consuming and fail to recapitulate the amyloid plaque and tau tangle simultaneously. The appearance of AD characteristic features in primary cell culture often requires several months^43–45^, and the time for detecting these phenotypes can be unpredictable, making data reproducibility a challenge. Thus, developing an efficient AD cellular model is urgent. Considering the overlapping cellular phenotypes between HGPS and AD and the induced expression of Lamin A in AD brains^8^, we propose that ectopic expression of progerin could be a useful strategy to promote an aging environment in the in vitro cellular models for better and faster modeling of AD.

To test this idea, we chose to modify one of the leading cellular models for AD, which requires several months to develop and display AD phenotypes documented by various publications^44,46^. We found that progerin expression could accelerate AD phenotype exhibition from 8–16 weeks to 3–4 weeks, providing a faster, cheaper, and more predictable platform for modeling AD.

## Results

### Different nuclear lamins are regulated differently during neural differentiation

We first characterized the expression of various lamins during the neural differentiation of ReN cells, a commercial human neural progenitor cell (NPC) line (Fig S1). By retracting growth factors from the medium, ReN cells generated neurite morphology (Fig S1a). To validate the cell types, we detected the cells with neuronal marker MAP2 and astrocytes marker GFAP by immunofluorescence staining. As expected, both markers were positively stained after two-week differentiation (Fig S1b). To further determine if these cells were functional, we stained the differentiated cells with a Ca2 + indicator, Fluo-4. By measuring the fluorescence change, the Ca2 + transients could be detected and therefore reflect the action potentials^47^. Cells at day 14 could generate Ca2 + transients regularly when the medium was replaced by Tyrode's solution supplemented with Na + (Fig S1c). This result suggested that ReN cells could generate action potentials after differentiation.

**Figure 1:**
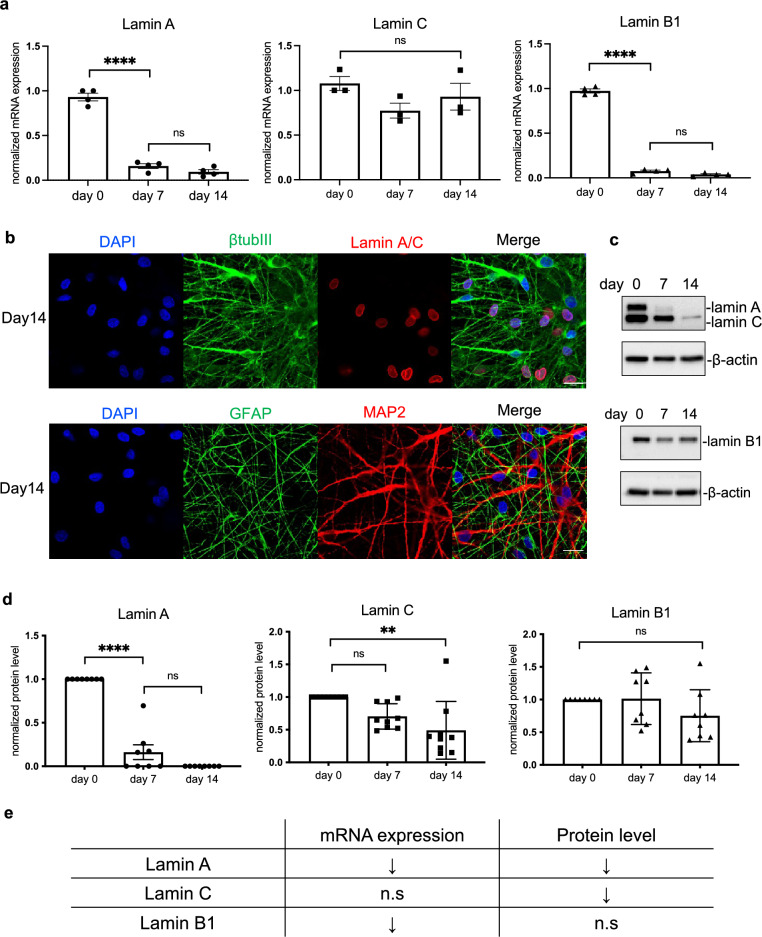
The expression of nuclear lamins mRNA and protein during the differentiation. (**a**) The quantification of mRNA relative expression of lamin A, lamin C and lamin B1 at different time points (day 0, day 7, day 14). Both lamin A and lamin B were significantly decreased while there was no significant change in lamin C mRNA. (**b**) Immunofluorescence staining of neuronal markers and lamin A/C at day 14. β-tubulin III and MAP2 are neuronal markers and GFAP is astrocytes markers. Lamin A/C staining is also positive in ReN cells after two-week differentiation. (Scale bar: 20 μm). (**c**,**d**) Western blot results of lamin A, lamin C and lamin B1 protein levels at different time points (day0, day7, day14). Both lamin A and lamin C were downregulated while lamin B1 was relatively stable. (**e**) Summary of the mRNA and protein expression pattern of nuclear lamins during NPC differentiation. All the results were generated from more than three biological replicates. Asterisks indicate statistical difference as follows: ns, not significant; *p < 0.05; **p < 0.01; ***p < 0.001; ****p < 0.0001.

To determine the expression pattern of nuclear lamins in neurons, we probed both transcriptional and translational expression of nuclear lamins during differentiation (Fig. 1). We found that at the mRNA level, both lamin A and lamin B1 were decreased, while lamin C did not show a significant change (Fig. 1a). However, at the protein level, lamin A and lamin C were downregulated, while lamin B1's protein level was relatively stable (Fig. 1c,d). These results (summarized in Fig. 1e) suggested that different nuclear lamins have different stability and different regulation mechanisms. While lamin A exists in the neural progenitor cells, it is diminished after the differentiation, which is consistent with previous findings^48^.

### Overexpression of lamin A and progerin results in neural death and cell cycle re-entry

Healthy mature neural cells prefer expressing lamin C rather than lamin A^48^. However, abnormal lamin A accumulation has been observed in patients' hippocampus through the different stages of AD^29,30^. To probe the potential role of A-type lamins in neurodegeneration, we overexpressed lamin A and progerin, respectively, in differentiating ReN cells to check their effects. Lentivirus constructs were used to express lamin A and progerin in ReN cells (Fig S2a), as previously described^49^. Both lamin A and progerin were tagged with EGFP^50^. Flow cytometry analysis indicated that transduction efficiencies were 71.1% and 64.8% in lamin A and progerin, respectively (Fig S2b,c). After transduction, ReN cells underwent the differentiation process. We checked the exogenous protein expression during the differentiation. By quantifying the protein level with Western blot (Fig S2d,e), we found that exogenous lamin A and progerin were still abundant after 2-week differentiation but exogenous progerin was decreased after 4 weeks. Thus, age-related phenotypes were detected at a 2-week timepoint.

**Figure 2:**
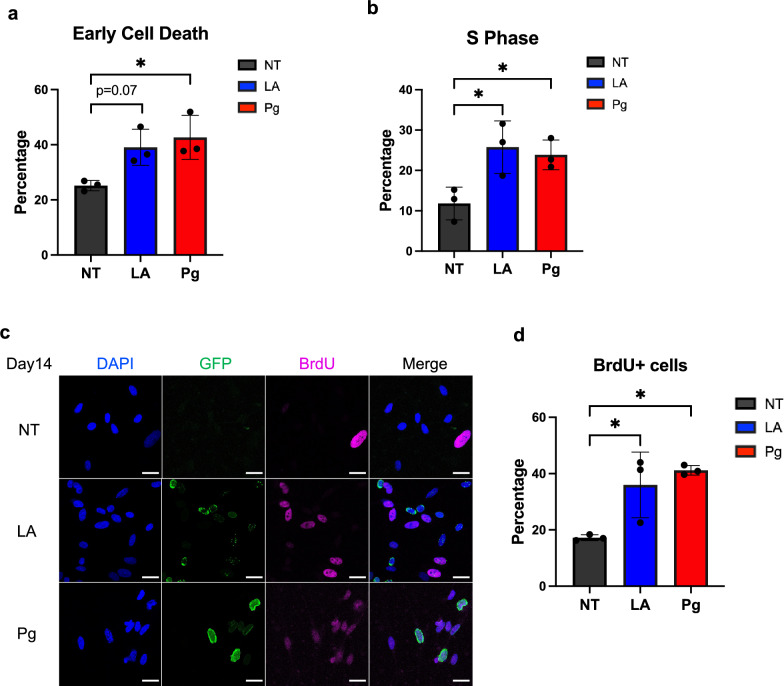
The effects of the overexpression of lamin A and progerin in neural cells. (**a**) ROS level after two-week lamin A and progerin overexpression. ROS level was significantly increased with progerin expression. (**b**) The quantification of cell death assay. The percentage of early cell death was significantly increased with progerin expression after two-week differentiation. (**c**) The quantification of cell cycle assay. The percentage of S-phase cells was increased after two-week lamin A- or progerin- transduction. (**d**,**e**) Immunofluorescence staining of BrdU in differentiated cells. Blue indicated DAPI signal, green indicated GFP-tagged lamin A or progerin, pink indicated BrdU signal. The percentage of BrdU positive cells over DAPI positive cells was increased after lamin A- or progerin- transduction. All the results were generated from three biological replicates. *p < 0.05. (Scale bar: 20 μm).

Cell death is a widely accepted feature of AD^51^, and we performed cell death flow cytometry to check this phenotype. After two weeks of differentiation, we observed a slight increase in the proportion of early cell death events in cells expressing lamin A compared to non-transduced cells (Fig. 2a, Fig S3a). Moreover, progerin-transduced cells showed a significant increase in cell death compared to non-transduced cells at the same time point. Although neurons are known to quit the cell cycle and stay in the quiescent stage (G0), cell cycle re-entry is considered an early event in neurodegeneration and is related to cell death^52,53^. A significantly higher percentage of S-phase cells was detected after 2-week lamin A-transduction, compared to the cells without any transductions (Fig. 2b, Fig S3b), which indicated increased cell cycle re-activation. We also stained the cells with BrdU to further validate the S-phase cells and confirmed that the percentage of S-phase cells was increased after 2 weeks (Fig. 2c,d). We observed nuclear morphology changes after progerin expression but not after lamin A expression (Fig S4), which indicated that progerin could disrupt the nucleoskeleton more drastically. In summary, the overexpression of lamin A led to heightened oxidative stress, cell death, and cell cycle re-entry, while progerin exacerbated these characteristics.

### The combination of progerin and FAD mutations accelerated AD hallmark phenotypes

As stated previously, the age clock is reset in iPSC-derived neurons even if the cells are from old donors^42^. In addition, in vitro cell culture often positively selects the more proliferative (i.e., younger and healthier) cells. Therefore, modeling an age-associated disease in vitro is tricky. Since we found that ectopic lamin A- or progerin- expression could induce age-associated phenotypes in ReN cells after 2-week differentiation (Fig. 2), we asked if lamin A- or progerin- expression could accelerate neurodegeneration in ReN cells with FAD mutations.

To test this idea, we adapted a well-characterized AD model^44^, which is the first cell culture model recapitulating ﻿both Aβ plaques and tau aggregations. The plasmid containing APP with both the K670N/M671L (Swedish) and V717I (London) mutations (APPSL) and PSEN1 with the Δ9 mutation (PSEN1(Δ9)) was utilized to introduce FAD mutants (Fig S5a), as described^44^. We transduced the ReN cells with lentivirus containing mcherry-tagged APPSL and PSEN1(Δ9) mutations (mAP) before the differentiation and used mcherry-only lentivirus as the control group. For 2D culture, cells were seeded on the Matrigel-coated plates. For 3D culture, the cell suspension was mixed with Matrigel with a certain ratio and then plated on the chamber slides. After 3 days, both APP mRNA and PSEN1 mRNA were increased after mAP transduction (Fig S5b). After a 2-week differentiation period, cells were transduced with either GFP-lamin A (LA) or GFP-progerin (Pg). Two days after transduction, both lamin A and progerin expression were robust. However, after 2 weeks, while lamin A expression remained strong, progerin expression became very weak (Fig S5c).

**Figure 3:**
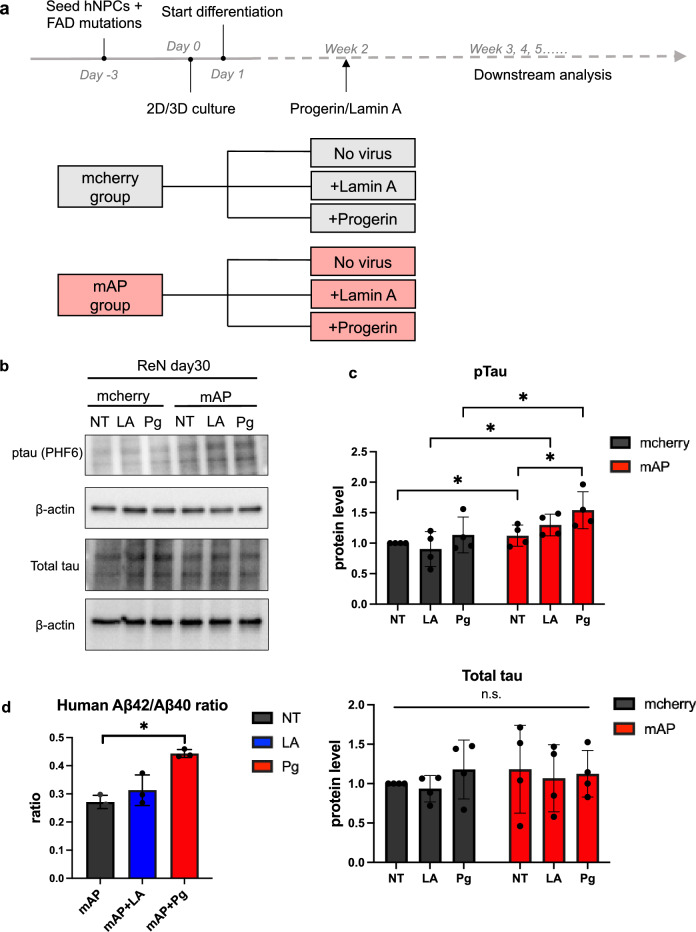

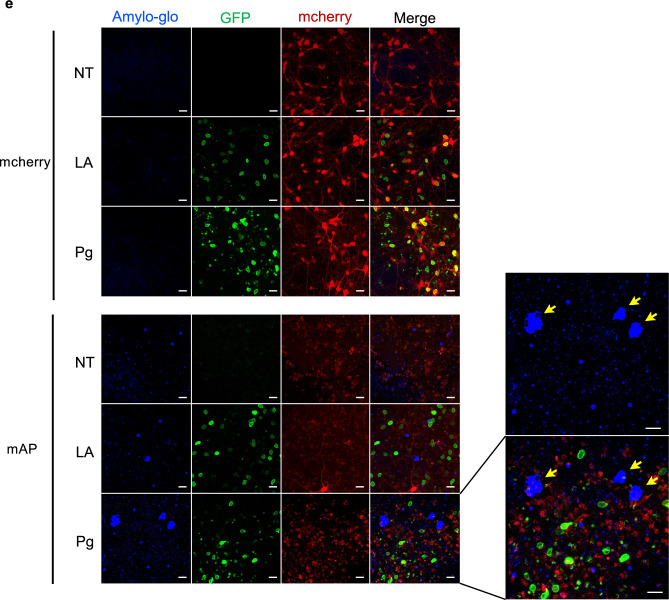
Accelerated AD protein features after FAD mutations and progerin co-transduction. (**a**) Experimental timeline. ReN cells were transduced with the lentivirus containing FAD mutation constructs before the differentiation. Cells were seeded on Matrigel-coated plates for 2D culture while cell suspension was mixed with Matrigel in 3D culture. After two-week differentiation, lamin A or progerin expression were transduced in the cells and followed by downstream analyses. Samples were as listed. (**b**,**c**) Protein level of total tau and phosphorylated tau after 4 weeks. The total tau level was not significantly changed. Phosphorylated tau was increased after lamin A expression, and progerin expression further upregulated tau phosphorylation significantly. Results were generated from four biological replicates. n.s., not significant; *p < 0.05. (**d**) Aβ42/ Aβ40 ratio after 3-week differentiation. Within the mAP group, Aβ42/ Aβ40 ratio was slightly increased after lamin A expression and significantly increased after progerin expression. Results were generated from three biological replicates. *p < 0.05. (**e**) Aβ aggregation staining with Amylo-glo after 4-week differentiation. Blue indicated the Amylo-glo staining, green indicated the GFP-tagged lamin A or progerin, red indicated mcherry or mcherry-tagged APP and PSEN1. Yellow arrows indicated the Aβ fibrils. (Scale bar: 20 μm).

Tau phosphorylation is an important hallmark of AD^18^. We used Western Blot to check the protein level of total tau and phosphorylated tau weekly. It is reported that the phosphorylation of Thr231 tau is an early event in AD among different phosphorylation sites^54^. It is estimated that ptau at Thr231 contributes about 26% of the overall inhibition of tau binding to microtubules^55^. Thus, an antibody that recognizes Thr231 phosphorylation site was used to detect tau phosphorylation. Although the total tau level was not significantly changed, ptau at Thr231 was slightly increased after lamin A expression compared to the cells with FAD mutations only, and progerin expression further upregulated tau phosphorylation significantly at week 4 (Fig. 3b,c).

Amyloid is another crucial pathology marker for AD^56^. A higher Aβ42/ Aβ40 ratio usually indicates more neurotoxicity, and it could be used as a more sensitive marker^17^. We used ELISA to detect the Aβ40 concentration, Aβ42 concentration, and Aβ42/ Aβ40 ratio in the culture medium after 3-week culture. Aβ40 level was upregulated in the mAP group after 3 weeks (Fig S5d). Aβ42 was barely detectable in the mcherry control group but was again upregulated in the mAP group after 3 weeks (Fig S5d). In the mAP group, the Aβ42/Aβ40 ratio showed a slight increase following the introduction of lamin A compared to cells carrying FAD mutants alone, and progerin expression significantly raised the Aβ42/Aβ40 ratio in comparison to cells with FAD mutants alone (Fig. 3d).

Next, we checked for any Aβ aggregation formations weekly in both 2D and 3D culture. In the 2D cell culture, we checked the cells with an Aβ oligomer antibody. In the mcherry group, Aβ oligomers were barely detected. In the mAP group, cells expressing both FAD mutations and progerin displayed stronger Aβ oligomer staining compared to cells expressing only FAD mutations after 3 weeks of culture (Fig S5e). Aβ usually diffuses into media in 2D cell culture and therefore it is difficult to detect fibril formation. To further visualize the aggregation, a 3D cell culture with Matrigel was adapted, and Amylo-glo was used to detect the Aβ fibrils weekly. Amylo-glo signals were first detected in the mAP group after four weeks of culture, whereas the signal remained negative in the mcherry control group (Fig. 3e). Notably, we observed larger Aβ fibrils in the mAP cells expressing both progerin and FAD mutations after four weeks, as compared to the cells expressing FAD mutations alone (Fig. 3e). These results indicated that ReN cells with the combination of progerin and FAD mutants displayed accelerated disease phenotypes after only 3–4 weeks in both 2D and 3D cell culture, including tau phosphorylation and formation of β-amyloid.

### The combination of progerin and FAD mutations leads to increasing cell cycle re-entry and increasing cell death

**Figure 4:**
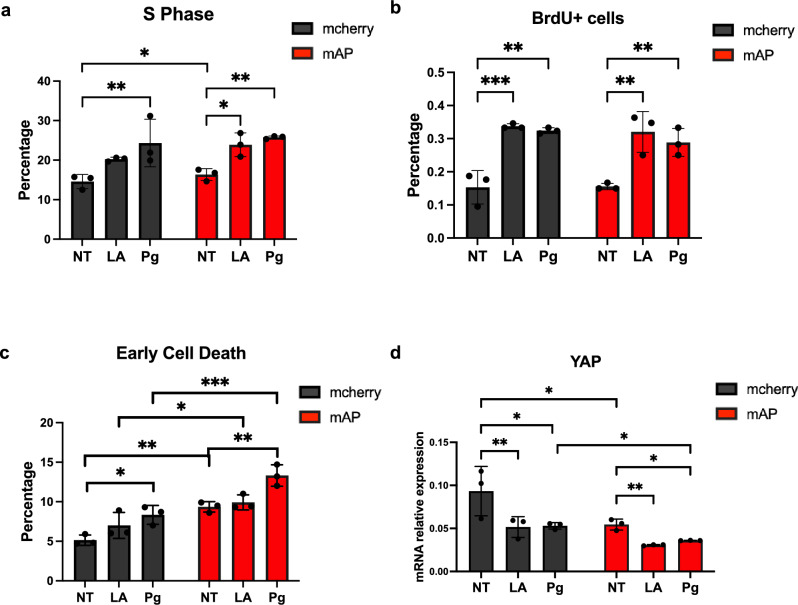
Increased cell cycle re-entry and cell death after FAD mutations and progerin co-transduction after 4 weeks. (**a**) The quantification of cell cycle assay. Within both mcherry control group and mAP group, S phase cells were increased after lamin A- and progerin- transduction. Comparing mAP cells to mcherry control cells, S phase percentage was increased as well. (**b**) The quantification of BrdU positive cells. An increased percentage of BrdU positive cells over DAPI positive cells after lamin A- and progerin- transduction was observed in both mcherry control group and mAP group. (**c**) The quantification of cell death flow cytometry. Cell death was increased comparing mAP group to mcherry control group. Within each group, progerin expression significantly induced more cell death. (**d**) The quantification of mRNA relative expression of YAP. YAP mRNA was downregulated in mAP ReN cells and progerin expression could further decrease YAP expression. All the results were generated from three biological replicates. n.s., not significant; *p < 0.05; **p < 0.01; ***p < 0.001; ****p < 0.0001.

After checking the AD pathological hallmarks, we investigated how cell cycle reactivation changed by adding lamin A or progerin. Overall, progerin addition resulted in more cell cycle re-entry events in 4 weeks (Fig. 4a). Within the mCherry group, ectopic expression of lamin A slightly increased the percentage of S-phase cells, and progerin could lead to a more drastic increase in S-phase cells after 4 weeks, compared to the cells with mCherry signal alone. Within the mAP group, cells exhibited a significantly higher percentage of S-phase cells after the ectopic expression of either lamin A or progerin. Meanwhile, a mild increasing percentage of S-phase cells was observed, comparing cells with FAD mutants alone to those with mCherry control plasmid. We further checked cell cycle re-entry with BrdU staining (Fig. 4b, S6). The number of BrdU-positive cells was significantly increased, comparing cells carrying FAD mutations with progerin to cells carrying FAD mutations without progerin (Fig. 4b, S6). The same trend could be observed for the combination of lamin A and FAD mutations.

It is reported that increased p16 and cdk4/6 are associated with cell cycle dysregulation in AD^57,58^. Thus, the translational expression of these cell cycle regulators was investigated. In general, the intervention of progerin gave rise to the significantly elevated mRNA level of p16 and cdk4/6 in both mcherry control group and mAP group in 4 weeks (Fig S7). Increased p16 level indicated that progerin could trigger senescence in cells and affect the environment. And cdk4/6 were involved in progerin-induced cell cycle reactivation. Ectopic expression of lamin A had the same trend (Fig S7). Although the transcriptional expressions of p16 and cdk4 were indistinguishable between cells with FAD mutants and cells with mcherry control plasmid (Fig S7a,b), cdk6 mRNA was significantly higher in cells with FAD mutants (Fig S7c).

Since cell cycle re-entry has been associated with cell death in neural cells^59^, we performed cell death flow cytometry. Results showed that progerin addition significantly induced more cell death events in both mcherry control group and mAP group in 4 weeks (Fig. 4c). Comparing each parallel sample in mAP group to the sample in mcherry control group, significantly increased cell death was detected as well (Fig. 4c). These results indicated that progerin and FAD mutants could have a synergetic effect in cell death.

Cell death is a critical event in AD progression^51^ and we would like to check the potential reasons in our system. Yes-associated protein (YAP) is a critical factor of the Hippo signaling pathway, which responds to changes in cell mechanics^60^. Several studies suggested.

YAP was downregulated in AD brains^61^ and it could be a critical regulator for cell death in AD^62^. Here we checked the translational expression of YAP. YAP mRNA was significantly downregulated in the cells with FAD mutants after 4 weeks, compared to the mcherry control group (Fig. 4d). The combination of progerin and FAD mutants exhibited a further reduction of YAP, compared to the cells with FAD mutants alone with 4-week culture (Fig. 4d). The accumulation of DNA damage could also contribute to cell death in AD^63^. Here we checked γ-H2AX expression as a marker for DNA damage. Ectopic expression of progerin could significantly upregulated γ-H2AX expression in both mAP group and mcherry control group in 4 weeks, whereas the difference between mcherry control group and mAP group was barely observed (Fig S8a,b). One possible explanation is that cells with FAD mutants alone might not be aged enough to develop detectable DNA damage. Meanwhile, it is reported that progerin could have a synergistic connection with telomere damage in cellular senescence^64^. Here we checked the telomere length in both mcherry group and mAP group. Telomere length was maintained after progerin overexpression (Fig S8c), which indicated progerin-induced aging in neurons is independent of telomere damage.

## Discussion

Starting from the nuclear lamina, via the trans-membrane LINC complex to the cytoskeleton filaments, the nucleoskeleton and the cytoskeleton form a network of physically interconnected cellular components^65^. The nuclear lamina plays a vital role in the signal transmission between the extracellular environment, cytoplasm, and nucleus^66^. During the aging process, age-related pathogenesis may occur due to changes in cellular mechanical properties resulting from disrupting the nucleocytoskeleton's integrity. This, in turn, could lead to dysfunctional changes. Protein aggregation is a common feature in most neurodegenerative diseases and could be linked to the disturbance in cell mechanics that occurs during the aging process. Meanwhile, laminopathies are mainly caused by mutations in the *LMNA* gene and manifest nuclear architecture disruption^67^. One of the laminopathies is HGPS, a premature aging disease^5^. Observations have revealed several similarities between premature aging diseases and physiological aging. These similarities include instability in both genomic and proteomic structures, an increase in oxidative stress, and impaired DNA repair mechanisms^68^. Additionally, in a drosophila model of AD, it was indicated that disruption of lamin led to relaxation of heterochromatin, activation of the cell cycle, and ultimately, cell death, all of which contribute to neurodegeneration^69^. Furthermore, numerous studies have demonstrated alterations in nuclear morphology and increased lamin A in individuals with Alzheimer's disease^27–30^. One group also observed reduced ZMPSTE24, a protein that plays a major role in the cleavage of the farnesylated tail in prelamin A, in patient’s brain^70^. These findings suggested that disrupted nuclear lamina could participate in AD pathology and farnesylated lamin A is associated.

Our study revealed that overexpression of lamin A in ReN cells resulted in elevated oxidative stress, reactivation of the cell cycle, and ultimately cell death. The presence of progerin, in particular, exacerbated these phenotypes, highlighting the potential role of lamin A in the development of AD pathology. As oxidative stress, cell cycle re-entry, and cell death are all crucial events in the aging process, these results suggest that the expression of lamin A or progerin may create an aging microenvironment conducive to the development of disease.

Currently, one of the biggest challenges in studying AD is accurately and efficiently modeling the disease. Considering the significant sequence differences in Aβ and tau between mice and humans, human tissue and cells could provide more accurate information^36,38^. However, a significant limitation in creating representative patient-derived models is the insufficient availability of high-quality post-mortem tissue. Consequently, most human-based models rely on induced pluripotent stem cells (iPSCs)^41^. Nonetheless, there are currently no standardized protocols for generating and maintaining these cell lines, and even in 3D cultures, it takes several months to observe AD phenotypes^44^. Additionally, a concern with iPSC-derived models is that the physiological age of the iPSCs may be reset, while AD is a late-onset disease. Thus, generating aged cells to study AD has become a pressing issue. Increased lamin A in AD patients might retain the farnesylated tail, given the observation that ZMPSTE24 is downregulated in AD brains^70^. And progerin is the permanently farnesylated lamin A because of the loss of the ZMPSTE24 cleavage site^5^. Moreover, HGPS exhibits molecular characteristics similar to those of natural aging, making it an effective model for aging research. Thus, using progerin to disrupt nuclear architecture could serve as a valuable strategy for emulating an aging environment. Progerin was already shown to induce age-related phenotypes in iPSC-derived neurons, successfully reproducing disease-specific phenotypes in Parkinson's Disease (PD)^71^. Ectopic expression of progerin was also applied in Huntington’s Disease (HD) and HD-associated gene profile changes were enhanced^72^.

Here we have put forward the idea that lamin A or progerin expression could create an environment conducive to the development of AD pathology. To test this hypothesis, we introduced exogenous lamin A or progerin into ReN cells containing FAD mutations to determine if AD-related features could be amplified. Our results demonstrated that ReN cells containing both progerin and FAD mutations exhibited a significant increase in the Aβ42/Aβ40 ratio and tau phosphorylation within just 3–4 weeks (Fig. 3). Additionally, Aβ aggregation was checked, and we observed stronger Aβ oligomer staining (Fig S4) and more Aβ fibril formation (Fig. 3e) in these cells after a 4-week culture period. We also found ectopic expression of either lamin A or progerin increased cell cycle re-entry events (Fig. 4a,b) and promoted cdk4/6 expression (Fig S7b, c), which are important cell cycle regulators. Furthermore, we noticed a significant increase in cell death after progerin expression (Fig. 4c), which are crucial events in neurodegeneration. Increased cell death could be an explanation for decreased progerin in 2 weeks after progerin transduction (Fig S5c). Meanwhile, we examined the senescence marker p16 and observed that both lamin A and progerin expression could significantly increase the mRNA level of p16, which supported that disrupted nucleoskeleton promoted senescence in cells and provided an aged environment for disease development (Fig S7a). Additionally, the presence of p16 indicated inappropriate regulation of neuronal cell cycle. Increased p16 level could be a response to cell cycle reactivation.

In summary, we only detected Aβ accumulations, increased tau phosphorylation and more cell death with the combination of ectopic progerin expression and FAD mutations. These findings suggest that progerin expression could accelerate the progression of AD-related phenotypes. No significant difference in cell cycle re-entry was observed between mcherry control group and mAP group. Considering AD is a late-onset disease, 4-week expression of FAD mutations might not be able to induce distinct changes in cell cycle dysregulation. Therefore, utilizing progerin strategy to facilitate AD progression is necessary.

**Figure 5:**
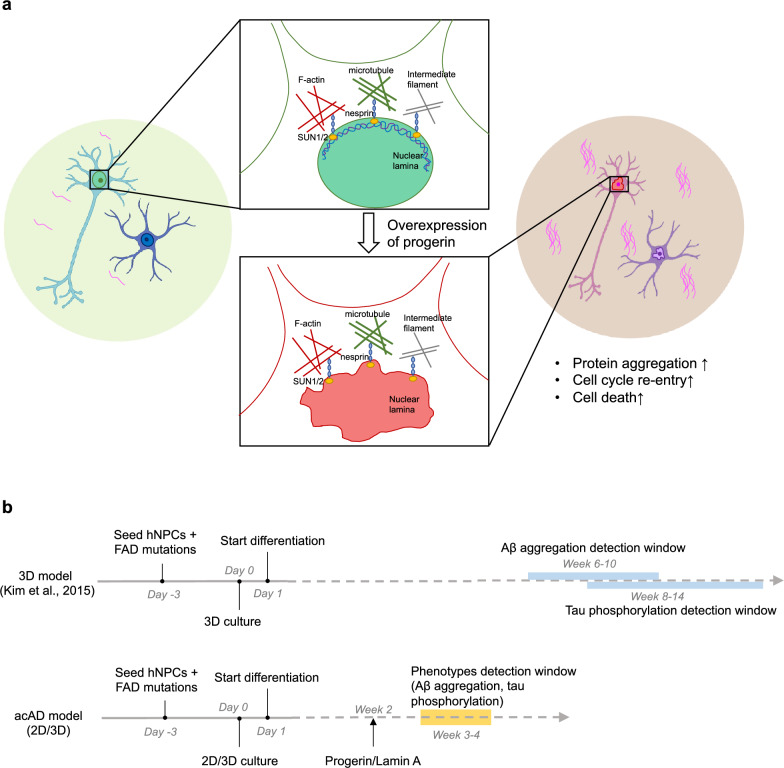
Proposed model of accelerated aging with progerin intervention. (**a**) We hypothesized that progerin could provide an aging environment for neurodegeneration. Cells containing only FAD mutations are not typically subject to aging in most circumstances. The connection between nucleoskeleton and cytoskeleton is integrated, and cell mechanic is well maintained. After progerin expression, the balance of the nucleoskeleton is disrupted and the cell environment could be stiffer, which makes cells vulnerable and results in more protein aggregation, cell cycle re-entry and cell death. (**b**) Comparison of schematic timeline between acAD model and a 3D model from Kim et al., 2015. Neural progenitor cells are differentiated from Week 0 in both protocols. Amyloid plaques and phosphorylated tau, two important AD hallmarks, were observed in the acAD model after 4-week differentiation, while it takes much longer in a well-characterized 3D AD model.

We hypothesize that cells containing only FAD mutations are not typically subject to aging in most circumstances. Therefore, their cell mechanics remain intact so they can clear toxic proteins. However, the balance of the nucleoskeleton is disrupted after progerin overexpression and progerin induces senescence in cells. And therefore it provides a stiff and aged microenvironment for neighboring cells, making cells more vulnerable and leading to increased protein aggregation, cell cycle re-entry, and cell death (Fig. 5a). In addition, it is known that progerin expression induces senescence in cells and promotes the secretion of senescent-associated secretory factors (SAPSs)^73,74^, which in turn affects the neighboring cells and creates an aged cell culture environment. Thus, we suggest that the introduction of progerin to the system acts both cell autonomously and non-autonomously, to induce aging and accelerate neurodegeneration.

Compared to the time-consuming animal models and rejuvenated iPSC-derived models, our system served as a more efficient model. This system was built on the well-characterized,

leading 3D AD cellular model^44^, and therefore we adapted their timeline for comparison (Fig. 5b). Our model demonstrated an accelerated manifestation of both increased tau phosphorylation and fibril formation within a significantly shorter timeframe of 4 weeks, in contrast to the traditional AD cellular models which typically require several months of experimental time. Meanwhile, traditional monolayer cell culture models fail to mimic the true brain architecture, resulting in Aβ diffusion in the culture medium^75^. Our system can be utilized in both 2D and 3D cultures, providing the ability to easily adjust cell density and extracellular matrix thickness for a variety of analyses. Consequently, this accelerated AD model may be a more feasible platform for drug screening and investigating AD mechanisms, including neuronal death. Our future work will compare the transcriptomic and proteomic profiles among different groups. These profiles will be used to further validate our model, aid us in exploring potential new pathways involved in AD, and identify early biomarkers of the disease.

To summarize, our study has established a link between lamin A and AD pathology. We have demonstrated that by inducing progerin expression in FAD-mutant cells, we can create an aged environment and generate strong AD features in a short amount of time (Fig. 5). This accelerated AD model could prove to be an effective tool for AD research. Furthermore, this progerin-induced aging can be used as a general approach for modeling other late-onset diseases.

## Materials and methods

### hNPC cell culture

ReNcell VM immortalized human neural progenitor cells (hNPC) were purchased from EMD Millipore, initially derived from the ventral mesencephalon region of human fetal brains. Cells were expanded in cell culture plates coated with Matrigel (Corning) and grown in BrainPhys Neuronal Medium (STEMCELL) containing 20 ng/ml bFGF (R&D Systems), 20 ng/ml EGF (Millipore Sigma), 10U/ml heparin (Sigma-Aldrich), B27 supplement (Thermo Fisher). For differentiation, cells were cultured in described medium lacking bFGF and EGF. For 3D cell culture on 4-chamber slides, 50ul cold Matrigel was added to 50ul cell suspension on ice. Matrigel/cell mixture was further diluted by adding 400ul of the cold ReN differentiation medium and then seeded on chamber slides. After overnight 37 °C incubation, 200ul of prewarmed ReN differentiation medium was added to each chamber.

### Lentivirus packaging and transduction

HEK293T cells (ATCC) were co-transfected with lentiviral plasmids and two virus packaging vectors, psPAX2 and pMD2.G (Addgene), utilizing Fugene 6 (Promega). Culture supernatants were collected at 48 h and 72 h post-transfection, and filtered through 0.45 μm filters to remove any nonadherent 293 T cells, then stored at − 80 °C. Next, ReN cells were infected by lentiviruses in media supplemented with Polybrene (Santa Cruz Biotechnology) with the final concentration of 8 μg/ml. The medium was changed every other day post-infection until the cells were harvested.

### RNA isolation and quantitative PCR

Total genomic RNA was extracted with Trizol (Life Technologies, Carlsbad, CA, USA) and purified using the RNeasy Mini kit (Qiagen, Hilden, Germany) as per the manufacturer's instructions. The RNA yield was determined by the NanoDrop 2000 spectrophotometer (Thermo Fisher Scientific, Waltham, MA, USA). 600 ng of total RNA was converted to cDNA using the iScript Select cDNA Synthesis kit (Bio-Rad). Quantitative RT-PCR was performed in triplicates using SYBR Green Supermix (Bio-Rad) on the CFX96 Real-Time PCR Detection System (C1000 Thermal Cycler, Bio-Rad). All primers used in this study are listed in Figure S9.

### Genomic DNA extraction and quantitative PCR telomere assay

DNA samples were extracted from ReN cells with PureLink™ Genomic DNA Mini Kit (Invitrogen). The mean telomere length was assessed by the modified monochrome multiplex quantitative polymerase chain reaction method (1). Relative telomere length is shown as T/S ratio, which stands for the ratio of telomere repeat copy number to single copy gene copy number. All primers used in this study are listed in Figure S9.

### Western blot

Whole-cell lysates for immunoblotting were prepared by dissolving cells in Laemmli Sample Buffer containing 5% 2-mercaptoethanol (Bio-Rad). Protein samples were loaded on 4–15% polyacrylamide gels (BioRad) and transferred onto 0.45 µm pore-size nitrocellulose membranes (Bio-Rad) using the Turboblot (BioRad). After that, blots were blocked with 5% milk for 1 h at room temperature. For phospho-tau, 5% BSA in TBS was used for blocking. Blots were incubated overnight at 4 °C with primary antibodies. And then blots were probed with secondary antibodies for 1 h at room temperature before ECL development and imaging (Bio-Rad). The primary antibodies used for immunoblotting are as follows: Lamin A/C antibody, (Abcam, 1:750); Lamin B1 antibody (Santa Cruz, 1:200); APP antibody (BioLegend, 1:400), total tau antibody (Santa Cruz, 1:200), PHF6 p-tau antibody (Santa Cruz, 1:200), γ-H2AX antibody (Abcam, 1:3000), and β-actin (1:5000, Sigma-Aldrich).

### Cell cycle assay

Cells were harvested with accutase and then washed with PBS. Next, ice-cold 70% ethanol was added to the cells and then cells were incubated at 4 °C for 1 h. After PBS wash, samples were treated with RNase at 37 °C for 30 min to remove RNA content. 5 μg Propidium Iodide (Invitrogen) was added to the samples for another 30 min incubation at 37 °C. Flow cytometry was performed with FACS CantoII (BD), and the data were analyzed by FlowJo software.

### Cell death assay

PI-annexin V apoptosis assay was performed according to the manufacturer's instruction (Thermo Fisher, A35122). In brief, cells were harvested and rinsed with PBS and then resuspended and stained with 100 μL of 1 × annexin V binding buffer, containing 5 μL of annexin V and 5 μL of PI, for 25 min in the dark at room temperature. Stained samples were analyzed by FACS CantoII (BD), and the data were processed by FlowJo software.

### Oxidative stress assay

Cellular ROS Assay kit (Abcam, ab186027) was used to check the oxidative stress according to the manufacturer's protocol. Cells were dissociated by accutase digestion, rinsed with PBS, and then incubated in 1 × ROS Red Stock Solution for 30 min at 37 °C. Flow cytometry was performed with FACS CantoII (BD), and the data were analyzed by FlowJo software.

### ELISA

Aβ40 and Aβ42 levels were mainly measured by Invitrogen amyloid-β human ELISA Kit (Thermo Fisher, KHB3481 and KHB3441) as per the manufacturer's protocol. The conditioned media from ReN cells were collected and diluted by 1:3 or 1:9 with a dilution buffer provided by the manufacturer. A plate reader (Thermo Scientific) was used to quantify Aβ40 and Aβ42 ELISA signals.

### Immunofluorescence staining

Cells were washed twice with PBS and then fixed in 4% paraformaldehyde (PFA) for 15 min at room temperature. After that, cells were permeabilized with 0.5% triton in PBS for 5 min at room temperature and then washed twice in TBS. Following blocking was done with 4% BSA in TBS for 1 h at room temperature. Samples were then incubated with primary antibodies in 4% BSA in TBS overnight at 4 °C. Primary antibodies were rinsed off with 5 washes of TBS. Samples were incubated with secondary antibodies in 4% BSA in TBS for 1 h at room temperature protected from light before being washed 5 times in TBS. Primary antibodies used include: MAP2 antibody (Abcam, 1:1000); β-tubIII antibody (Abcam, 1:1000); GFAP antibody (Cell Signaling, 1:1000); Lamin A/C antibody, (Abcam, 1:500); Lamin B1 antibody (Santa Cruz, 1:200). Secondary antibodies include: Alexa Fluor 488 donkey anti-rabbit IgG (1:1000, Invitrogen), Alexa Fluor 594 donkey anti-rabbit IgG (1:1000, Invitrogen), Alexa Fluor 488 donkey anti-mouse IgG (1:1000, Invitrogen), Alexa Fluor 594 donkey anti-mouse IgG (1:1000, Invitrogen) and Alexa Fluor 644 donkey anti-rabbit IgG (1:1000, Invitrogen).

For BrdU staining, cells were incubated in a 10 uM BrdU (BD #550,891) labeling medium for 12 h. Cells were then washed with PBS, fixed and permeabilized. Afterward, cells were denatured in 2N HCl for 40 min at room temperature. Next, samples were incubated in Alexa Fluor 647 anti-BrdU antibody solution (1:1000, Invitrogen #B35133) at 4 °C overnight. Samples were protected and stained in vectashield mounting medium with DAPI (Vector) sealed with a coverslip and stored in the dark. Fluorescence images were acquired with a Zeiss LSM 710 confocal microscope (Zeiss International, Oberkochen, Germany).

### Amylo-glo staining

﻿Cells were washed three times with 0.9% (wt/vol) NaCl solution. Following adding 100 µl of 0.05 × Amylo-Glo working solution, 3D culture cells were incubated for 5 min at room temperature. And then, the staining solution was removed. 200 µl of 0.9% saline was added and followed with 5-min incubation. Samples were washed three times with ddH2O, and further washed with 0.9% (wt/vol) NaCl solution three times. Samples were protected in antifade vectashield mounting medium without DAPI (Vector) and stored in the dark. Fluorescence images were acquired with a Zeiss LSM 710 confocal microscope (Zeiss International, Oberkochen, Germany).

### Calcium preparation and imaging

Intracellular calcium labeling was prepared using Fluo-4 AM (Thermo Fisher Scientific) following manufacturer-provided protocols. Briefly, a 1 mM stock solution was prepared in anhydrous DMSO. Cells were incubated in standard cell specific medium for 1 h at 37 °C with 1uM Fluo-4 AM. After incubation cells were rinsed with DPBS and then the fresh, appropriate medium was added. Cell samples were then immediately used for imaging using 488 nm laser to excite the Fluo-4 AM dye.

### Data analysis

Statistical analyses were performed using GraphPad Prism 7 software. Data were analyzed using unpaired Student's *t*-test for two groups. One-way and two-way analysis of variance (ANOVA) followed by post hoc multiple comparisons were used to compare the means of three or more groups. All experiments were repeated at least three times, and the results are presented as the mean ± SD. A *p* value < 0.05 was considered significant. Asterisks indicate statistical difference as follows: n.s., not significant; *p < 0.05; **p < 0.01; ***p < 0.001; ****p < 0.0001.

### Supplementary Information

Supplementary Legends.

Supplementary Figures.

## Data Availability

The datasets used and/or analyzed during the current study available from the corresponding author on reasonable request.

## References

[1] Bridger JM, Foeger N, Kill IR, Herrmann H (2007). The nuclear lamina. FEBS J..

[2] Lin F, Worman HJ (1993). Structural organization of the human gene encoding nuclear lamin A and nuclear lamin C. J. Biol. Chem..

[3] Lin F, Worman HJ (1995). Structural organization of the human gene (LMNB1) encoding nuclear lamin B1. Genomics.

[4] Biamonti G (1992). The gene for a novel human lamin maps at a highly transcribed locus of chromosome 19 which replicates at the onset of S-phase. Mol. Cell. Biol..

[5] Eriksson M (2003). Recurrent de novo point mutations in lamin A cause Hutchinson–Gilford progeria syndrome. Nature.

[6] Goldman RD (2004). Accumulation of mutant lamin A causes progressive changes in nuclear architecture in Hutchinson-Gilford progeria syndrome. Proc. Natl. Acad. Sci. USA.

[7] McCord RP (2013). Correlated alterations in genome organization, histone methylation, and DNA–lamin A/C interactions in Hutchinson-Gilford progeria syndrome. Genome Res..

[8] Frost B (2016). Alzheimer’s disease: An acquired neurodegenerative laminopathy. Nucleus.

[9] Erkkinen MG, Kim MO, Geschwind MD (2018). Clinical neurology and epidemiology of the major neurodegenerative diseases. Cold Spring Harb. Perspect. Biol..

[10] Bekris LM, Yu C-E, Bird TD, Tsuang DW (2010). Genetics of Alzheimer disease. J. Geriatr. Psychiatry Neurol..

[11] Serrano-Pozo A, Frosch MP, Masliah E, Hyman BT (2011). Neuropathological alterations in Alzheimer disease. Cold Spring Harb. Perspect. Med..

[12] Gauthier S, Webster C, Servaes S, Morais JA, R.-N. P. World Alzheimer Report 2022: Life after diagnosis: Navigating treatment, care and support. *Alzheimer’s Dis. Int.* **25**, 50 (2022).

[13] O’Brien RJ, Wong PC (2011). Amyloid precursor protein processing and Alzheimer’s disease. Annu. Rev. Neurosci..

[14] Karran E, Mercken M, De Strooper B (2011). The amyloid cascade hypothesis for Alzheimer’s disease: An appraisal for the development of therapeutics. Nat. Rev. Drug Discov..

[15] Kim J (2007). Aβ40 inhibits amyloid deposition in vivo. J. Neurosci..

[16] Findeis MA (2007). The role of amyloid β peptide 42 in Alzheimer’s disease. Pharmacol. Ther..

[17] Kuperstein I (2010). Neurotoxicity of Alzheimer’s disease Aβ peptides is induced by small changes in the Aβ42 to Aβ40 ratio. EMBO J..

[18] Kolarova M, García-Sierra F, Bartos A, Ricny J, Ripova D (2012). Structure and pathology of tau protein in Alzheimer disease. Int. J. Alzheimers. Dis..

[19] Lippens G (2007). Tau aggregation in Alzheimer’s disease: What role for phosphorylation?. Prion.

[20] Neddens J (2018). Phosphorylation of different tau sites during progression of Alzheimer’s disease. Acta Neuropathol. Commun..

[21] Ittner LM, Götz J (2011). Amyloid-β and tau—a toxic pas de deux in Alzheimer’s disease. Nat. Rev. Neurosci..

[22] Bloom GS (2014). Amyloid-β and tau: The trigger and bullet in Alzheimer disease pathogenesis. JAMA Neurol..

[23] Kametani, F. & Hasegawa, M. Reconsideration of amyloid hypothesis and tau hypothesis in Alzheimer’s disease. *Front. Neurosci.* **12**, (2018). 10.3389/fnins.2018.00025 PMC5797629 29440986

[24] Kadowaki H (2004). Amyloid β induces neuronal cell death through ROS-mediated ASK1 activation. Cell Death Differ..

[25] Seward ME (2013). Amyloid-β signals through tau to drive ectopic neuronal cell cycle re-entry in alzheimer’s disease. J. Cell Sci..

[26] Zhang H, Cao Y, Ma L, Wei Y, Li H (2021). Possible mechanisms of tau spread and toxicity in Alzheimer’s disease. Front. Cell Dev. Biol..

[27] Sheffield LG, Miskiewicz HB, Tannenbaum LB, Mirra SS (2006). Nuclear pore complex proteins in Alzheimer disease. J. Neuropathol. Exp. Neurol..

[28] Frost B, Hemberg M, Lewis J, Feany MB (2014). Tau promotes neurodegeneration through global chromatin relaxation. Nat. Neurosci..

[29] Méndez-López, I. *et al.* Hippocampal LMNA gene expression is increased in late-stage alzheimer’s disease. *Int. J. Mol. Sci.* **20**, (2019). 10.3390/ijms20040878 PMC6413092 30781626

[30] Gil L (2020). Perinuclear lamin A and nucleoplasmic lamin B2 characterize two types of hippocampal neurons through Alzheimer’s disease progression. Int. J. Mol. Sci..

[31] Wang L (2018). Tissue and cellular rigidity and mechanosensitive signaling activation in Alexander disease. Nat. Commun..

[32] Xiong, Z. M., LaDana, C., Wu, D. & Cao, K. An inhibitory role of progerin in the gene induction network of adipocyte differentiation from iPS cells. *Aging (Albany. NY).* **5**, 288–303 (2013). 10.18632/aging.100550 PMC3651521 23596277

[33] Cummings JL, Morstorf T, Zhong K (2014). Alzheimer’s disease drug-development pipeline: Few candidates, frequent failures. Alzheimers. Res. Ther..

[34] Cummings JL, Morstorf T, Zhong K (2014). Alzheimer’s disease drug-development pipeline: Few candidates, frequent failures. Alzheimer’s Res. Ther..

[35] Banik A (2015). Translation of pre-clinical studies into successful clinical trials for Alzheimer’s disease: What are the roadblocks and how can they be overcome?. J. Alzheimer’s Dis..

[36] Xu G (2015). Murine Aβ over-production produces diffuse and compact Alzheimer-type amyloid deposits. Acta Neuropathol. Commun..

[37] Drummond E, Wisniewski T (2017). Alzheimer’s disease: Experimental models and reality. Acta Neuropathol..

[38] Duff K (2000). Characterization of pathology in transgenic mice over-expressing human genomic and cDNA tau transgenes. Neurobiol. Dis..

[39] Andorfer C (2003). Hyperphosphorylation and aggregation of tau in mice expressing normal human tau isoforms. J. Neurochem..

[40] Xia D (2022). Novel App knock-in mouse model shows key features of amyloid pathology and reveals profound metabolic dysregulation of microglia. Mol. Neurodegener..

[41] Penney J, Ralvenius WT, Tsai L-H (2020). Modeling Alzheimer’s disease with iPSC-derived brain cells. Mol. Psychiatry.

[42] Lapasset L (2011). Rejuvenating senescent and centenarian human cells by reprogramming through the pluripotent state. Genes Dev..

[43] Shi, Y. *et al.* A human stem cell model of early Alzheimer’s disease pathology in Down syndrome. *Sci. Transl. Med.* **4**, 124ra29 (2012). 10.1126/scitranslmed.3003771 PMC4129935 22344463

[44] Kim YH (2015). A 3D human neural cell culture system for modeling Alzheimer’s disease. Nat. Protoc..

[45] Jorfi, M., D’Avanzo, C., Tanzi, R. E., Kim, D. Y. & Irimia, D. Human neurospheroid arrays for in vitro studies of alzheimer’s disease. *Sci. Rep. 8*, 1–13 (2018). 10.1038/s41598-018-20436-8 PMC5799361 29402979

[46] Choi SH (2014). A three-dimensional human neural cell culture model of Alzheimer’s disease. Nature.

[47] Grienberger C, Konnerth A (2012). Imaging calcium in neurons. Neuron.

[48] Jung HJ (2012). Regulation of prelamin A but not lamin C by miR-9, a brain-specific microRNA. Proc. Natl. Acad. Sci. USA.

[49] Wu D, Yates PA, Zhang H, Cao K (2016). Comparing lamin proteins post-translational relative stability using a 2A peptide-based system reveals elevated resistance of progerin to cellular degradation. Nucleus.

[50] Wu D, Flannery AR, Cai H, Ko E, Cao K (2014). Nuclear localization signal deletion mutants of lamin A and progerin reveal insights into lamin A processing and emerin targeting. Nucleus.

[51] Goel P (2022). Neuronal cell death mechanisms in Alzheimer’s disease: An insight. Front. Mol. Neurosci..

[52] Kruman II (2004). Cell cycle activation linked to neuronal cell death initiated by DNA damage. Neuron.

[53] Barrio-Alonso E, Hernández-Vivanco A, Walton CC, Perea G, Frade JM (2018). Cell cycle reentry triggers hyperploidization and synaptic dysfunction followed by delayed cell death in differentiated cortical neurons. Sci. Rep..

[54] Luna-Muñoz J (2005). Regional conformational change involving phosphorylation of tau protein at the Thr 231, precedes the structural change detected by Alz-50 antibody in Alzheimer’s disease. J. Alzheimer’s Dis..

[55] Sengupta A (1998). Phosphorylation of tau at both Thr 231 and Ser 262 is required for maximal inhibition of its binding to microtubules. Arch. Biochem. Biophys..

[56] Chen GF (2017). Amyloid beta: Structure, biology and structure-based therapeutic development. Acta Pharmacol. Sin..

[57] Mcshea A (1997). Abnormal expression of the cell cycle regulators P16 and CDK4 in Alzheimer’s disease. Am. J. Pathol..

[58] Bhat R (2012). Astrocyte senescence as a component of Alzheimer’s disease. PLoS ONE.

[59] Lee HG (2009). Cell cycle re-entry mediated neurodegeneration and its treatment role in the pathogenesis of Alzheimer’s disease. Neurochem. Int..

[60] Piccolo S, Dupont S, Cordenonsi M (2014). The biology of YAP/TAZ: Hippo signaling and beyond. Physiol. Rev..

[61] Xu X (2021). YAP prevents premature senescence of astrocytes and cognitive decline of Alzheimer’s disease through regulating CDK6 signaling. Aging Cell.

[62] Tanaka H (2020). YAP-dependent necrosis occurs in early stages of Alzheimer’s disease and regulates mouse model pathology. Nat. Commun..

[63] Madabhushi R, Pan L, Tsai LH (2014). DNA damage and its links to neurodegeneration. Neuron.

[64] Cao K (2011). Progerin and telomere dysfunction collaborate to trigger cellular senescence in normal human fibroblasts. J. Clin. Investig..

[65] Houben F, Ramaekers FCS, Snoeckx LHEH, Broers JLV (2007). Role of nuclear lamina-cytoskeleton interactions in the maintenance of cellular strength. Biochim. Biophys. Acta Mol. Cell Res..

[66] Gerace L, Tapia O (2018). Messages from the voices within: Regulation of signaling by proteins of the nuclear lamina. Curr. Opin. Cell Biol..

[67] Worman HJ (2012). Nuclear lamins and laminopathies. J. Pathol..

[68] Kubben N, Misteli T (2017). Shared molecular and cellular mechanisms of premature ageing and ageing-associated diseases. Nat. Rev. Mol. Cell Biol..

[69] Frost B, Bardai FH, Feany MB (2016). Lamin dysfunction mediates neurodegeneration in tauopathies. Curr. Biol..

[70] Rosene MJ (2021). LMNA-mediated nucleoskeleton dysregulation in Alzheimer disease. Alzheimers. Dement..

[71] Miller JD (2013). Human iPSC-based modeling of late-onset disease via progerin-induced aging. Cell Stem Cell.

[72] Cohen-Carmon D (2020). Progerin-induced transcriptional changes in huntington’s disease human pluripotent stem cell-derived neurons. Mol. Neurobiol..

[73] Bidault G (2020). Progerin expression induces inflammation, oxidative stress and senescence in human coronary endothelial cells. Cells.

[74] Xu Q (2022). Vascular senescence in progeria: Role of endothelial dysfunction. Eur. Hear. J. Open.

[75] Centeno EGZ, Cimarosti H, Bithell A (2018). 2D versus 3D human induced pluripotent stem cell-derived cultures for neurodegenerative disease modelling. Mol. Neurodegener..

