# Supplementary material for: Development of an accelerated cellular model for early changes in Alzheimer’s disease
Source: Sci Rep. 2023 Oct 26;13:18384. doi: 10.1038/s41598-023-45826-5 (PMC10603068; doi:10.1038/s41598-023-45826-5)
Supplement: Supplementary file 1 — Supplementary Legends. [file 41598_2023_45826_MOESM1_ESM.docx]

**Development of an Accelerated Cellular Model for early changes in Alzheimer's Disease**

Huijing Xue^1^, Sylvester Gate III^2^, Emma Gentry^1^, Wolfgang Losert^2^, and Kan Cao^1,*^

1. Department of Cell Biology and Molecular Genetics, University of Maryland, College Park, MD 20742, USA

2. Institute of Physical Sciences, University of Maryland, College Park, MD 20742, USA

**Supplementary figure legends**

**Figure S1. The process of ReN cell differentiation.**

(a) ReN cells could grow neurites in two weeks. (Scale bar: 100um)

(b) Differentiated cells were positive with neuronal markers, MAP2 and astrocyte markers, GFAP. (Scale bar: 20um)

(c) Differentiated cells could generate Ca2+ transients in the Tyrode's solution supplemented with Na+.

**Figure S2. Lamin A- and progerin- transduction in ReN cells.**

(a) Diagrams of lentiviral constructs. GFP-LA virus contains lamin A cDNA and GFP-Pg virus contains progerin cDNA. Both vectors contain EGFP signal.

(b)(c) Quantification of transduction efficiency. The transduction was successful, and the efficiency was 71.1% for GFP-LA virus and 64.8% for GFP-Pg virus after 2-day transduction.

(d)(e) Western blot results of exogenous protein level and GFP signals during the differentiation. Exogenous progerin protein were decreased during the differentiation.

**Figure S3. Representative flow cytometry plots.**

(a) Annexin V/PI flow cytometry plots. (b) Cell cycle flow cytoetry analysis with Dean-Jett-Fox model.

**Figure S4. Nuclear morphology changes after progerin transduction.**

Abnormal nuclear morphology was only observed after progerin expression but not after lamin A expression. White arrows point to the nucleus with severe nuclear morphology changes. (Scale bar: 20μm)

**Figure S5. Combination of FAD mutations and progerin-transduction in ReN cells**

(a) Diagrams of lentiviral constructs. A plasmid containing APP with both the K670N/M671L (Swedish) and V717I (London) mutations (APPSL) and PSEN1 with the Δ9 mutation (PSEN1(Δ9)) was a gift from Dr. Kim's lab.

(b) Quantification of mRNA level after FAD transduction. The transcription level of both APP and PSEN1 was upregulated after the transduction.

(c) Western blot of exogenous lamin A and progerin. After 2-day transduction, both lamin A and progerin expression were abundant. After 2 weeks, lamin A level was still abundant, while progerin level became very weak.

(d) Protein concentration of Aβ40 and 42 in the culture medium after 3-week culture.

**Figure S6. Aβ oligomer staining after 3-week differentiation.**

Pink indicated Aβ oligomer staining, red indicated mcherry signal, green indicated the GFP-tagged lamin A or progerin, blue indicated DAPI signal (Scale bar: 20μm).

**Figure S7. The mRNA expression of cell-cycle-related regulators after 4 weeks.**

(a). Quantification of p16 mRNA expression. Within mcherry and mAP groups, p16 mRNA level was significantly increased after lamin A expression. Same trend was observed after progerin expression. Results were generated from four biological replicates. n.s., not significant; *p < 0.05; **p < 0.01; ***p < 0.001; ****p < 0.0001.

(b). Quantification of cdk4 mRNA expression. Within mcherry and mAP groups, cdk4 mRNA level was significantly increased after lamin A expression. Same trend was observed after progerin expression. Results were generated from three biological replicates. n.s., not significant; *p < 0.05; **p < 0.01; ***p < 0.001; ****p < 0.0001.

(c). Quantification of cdk6 mRNA expression. mRNA level of cdk6 was higher in the cells with FAD mutants alone than the cells with mcherry control alone. Within mcherry and mAP groups, cdk6 mRNA level was significantly increased after lamin A expression. Same trend was observed after progerin expression. Results were generated from three biological replicates. n.s., not significant; *p < 0.05; **p < 0.01; ***p < 0.001; ****p < 0.0001.

**Figure S8. DNA damage and telomere length after progerin addition.**

(a)(b). Western blot of γH2AX. γ-H2AX was upregulated after ectopic lamin A expression in both mcherry control group and mAP group after 4 weeks. Same trend was observed after progerin expression. Results were generated from three biological replicates. n.s., not significant; *p < 0.05; **p < 0.01; ***p < 0.001; ****p < 0.0001.

(c). Quantification of telomere length with qPCR after 4 weeks. Telomere length did not show significant change with FAD intervention or progerin intervention. Results were generated from three biological replicates. n.s., not significant.
