# Supplementary material for: Development of an accelerated cellular model for early changes in Alzheimer’s disease
Source: Sci Rep. 2023 Oct 26;13:18384. doi: 10.1038/s41598-023-45826-5 (PMC10603068; doi:10.1038/s41598-023-45826-5)
Supplement: Supplementary file 2 — Supplementary Figures. [file 41598_2023_45826_MOESM2_ESM.pdf]

Development of an Accelerated Cellular Model for early changes in Alzheimer's Disease

Huijing Xue[1], Sylvester Gate III[2], Emma Gentry[1], Wolfgang Losert[2], and Kan Cao[1,*]

1. Department of Cell Biology and Molecular Genetics, University of Maryland, College Park, MD 20742, USA

2. Institute of Physical Sciences, University of Maryland, College Park, MD 20742, USA

*: Corresponding author

Dr. Kan Cao

Email: kcao@umd.edu

Figure S1.

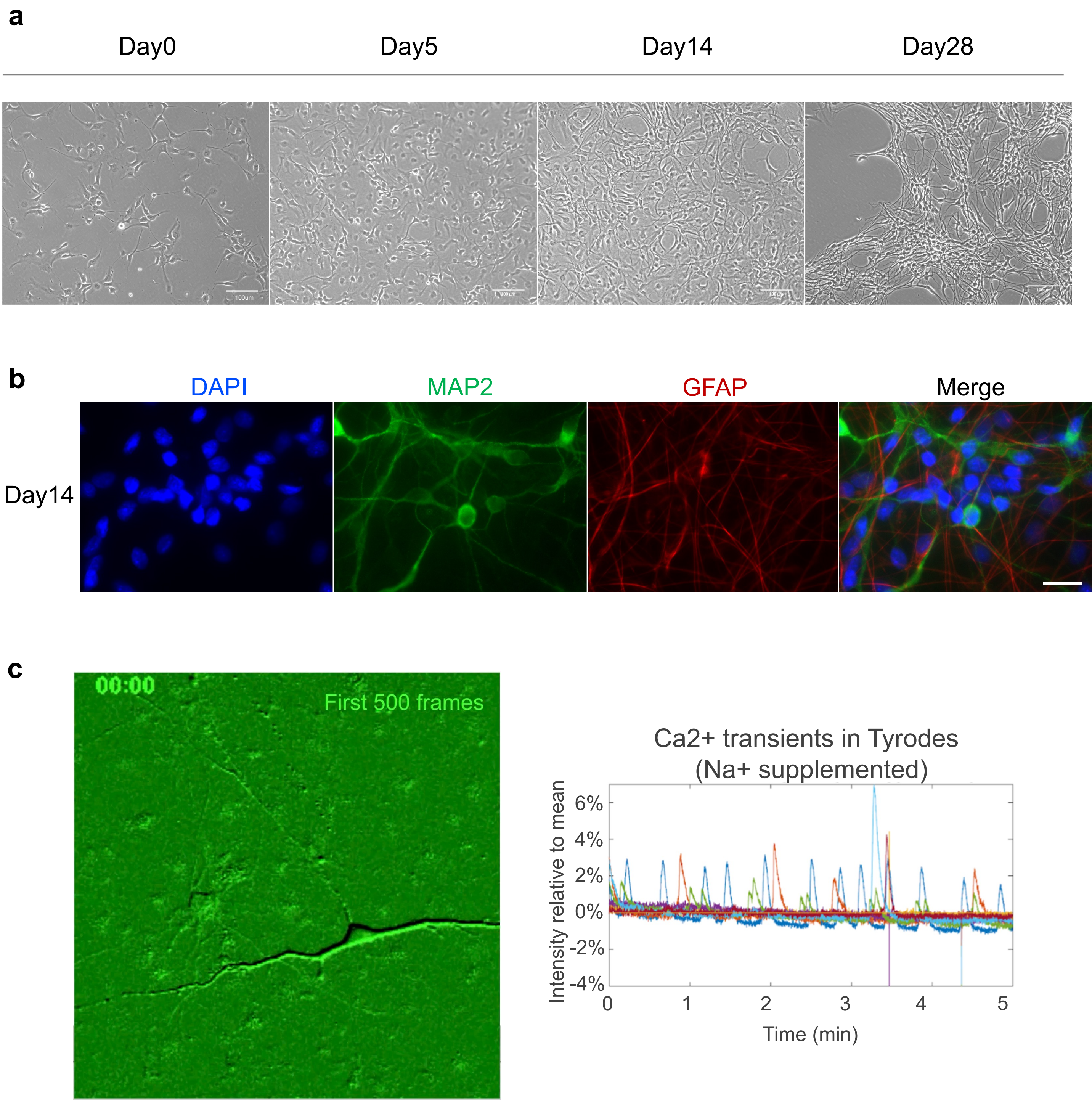

Figure S1. The process of ReN cell differentiation.
(a) ReN cells could grow neurites in two weeks. (Scale bar: 100um)
(b) Differentiated cells were positive with neuronal markers, MAP2 and astrocyte markers, GFAP. (Scale bar: 20um)
(c) Differentiated cells could generate Ca2+ transients in the Tyrode's solution supplemented with Na+.

Figure S2.

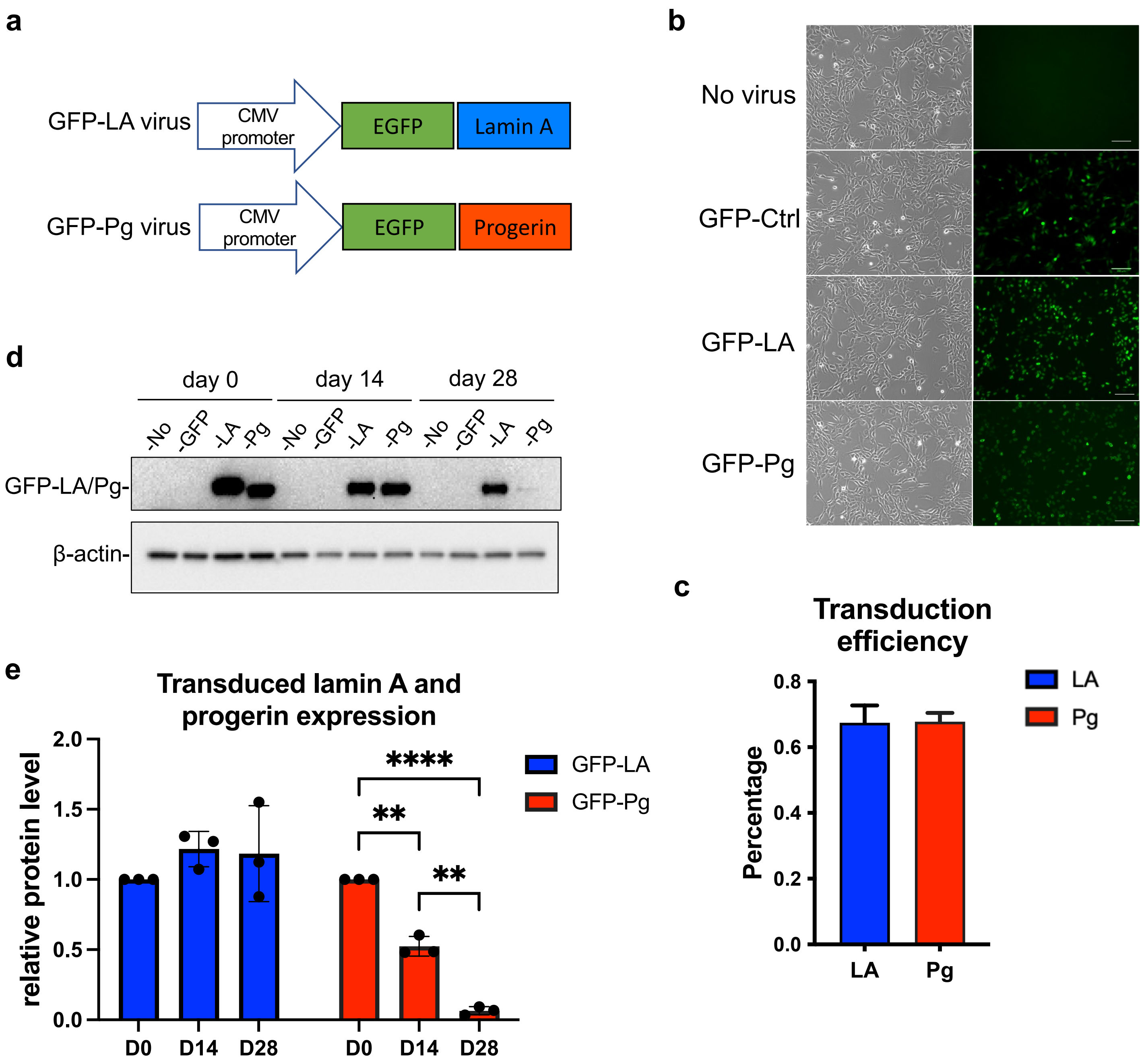
a
GFP-LA virus
CMV promoter
EGFP
Lamin A
GFP-Pg virus
CMV promoter
EGFP
Progerin
b
No virus
GFP-Ctrl
GFP-LA
GFP-Pg
c
Transduction efficiency
Percentage
0.0
0.2
0.4
0.6
0.8
LA
Pg
d
day 0
day 14
day 28
-No
-GFP
-LA
-Pg
GFP-LA/Pg-
β-actin-
e
Transduced lamin A and progerin expression
relative protein level
0.0
0.5
1.0
1.5
2.0
D0
D14
D28
GFP-LA
GFP-Pg
****
**
**

Figure S3.

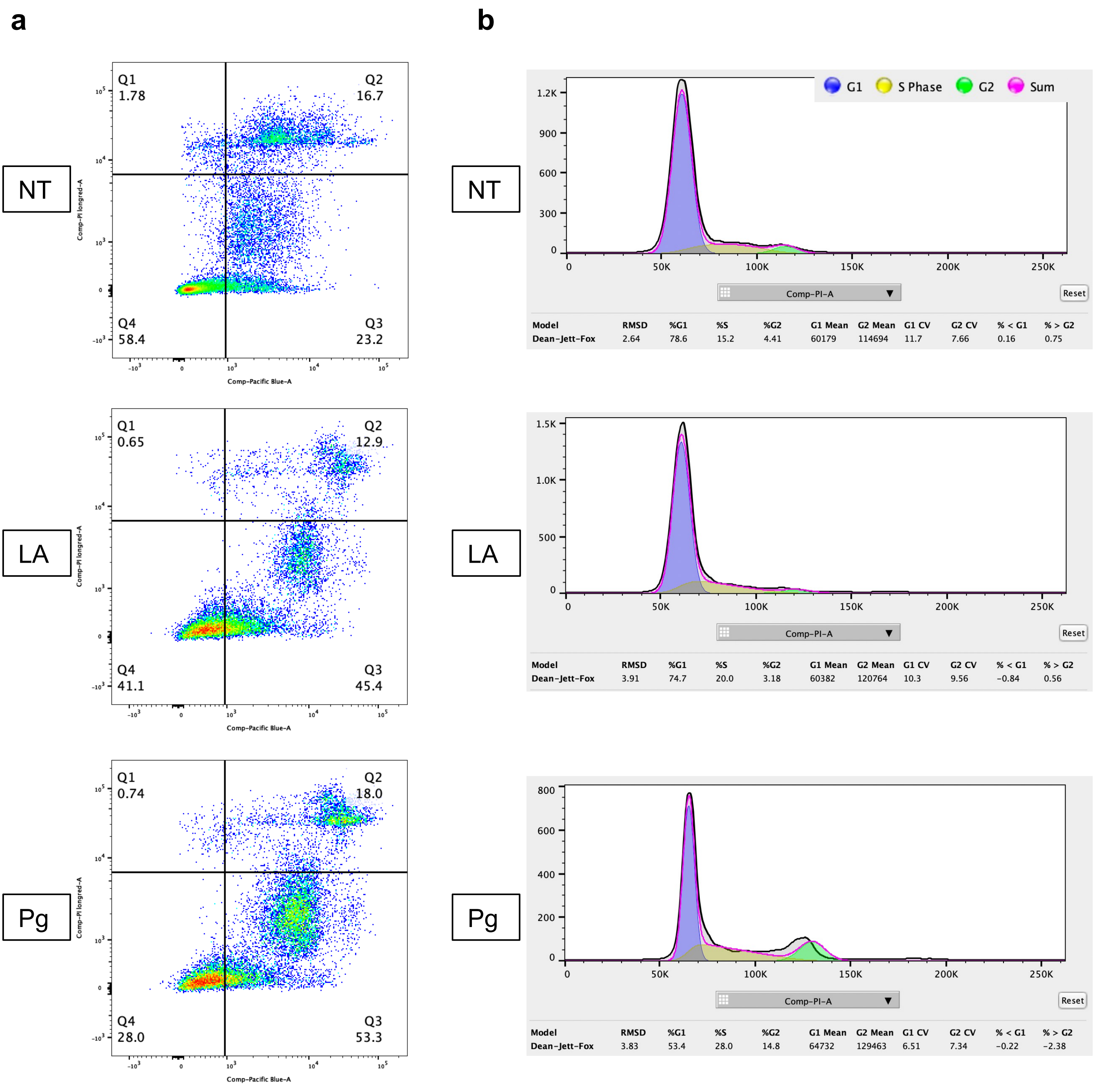
a
b
NT
LA
Pg
Q1 1.78
Q2 16.7
Q4 58.4
Q3 23.2
Q1 0.65
Q2 12.9
Q4 41.1
Q3 45.4
Q1 0.74
Q2 18.0
Q4 28.0
Q3 53.3
Comp-PI longred-A
Comp-Pacific Blue-A
G1
S Phase
G2
Sum
Comp-PI-A
Reset
Model RMSD %G1 %S %G2 G1 Mean G2 Mean G1 CV G2 CV % < G1 % > G2
Dean-Jett-Fox 2.64 78.6 15.2 4.41 60179 114694 11.7 7.66 0.16 0.75
Dean-Jett-Fox 3.91 74.7 20.0 3.18 60382 120764 10.3 9.56 -0.84 0.56
Dean-Jett-Fox 3.83 53.4 28.0 14.8 64732 129463 6.51 7.34 -0.22 -2.38

Figure S4.

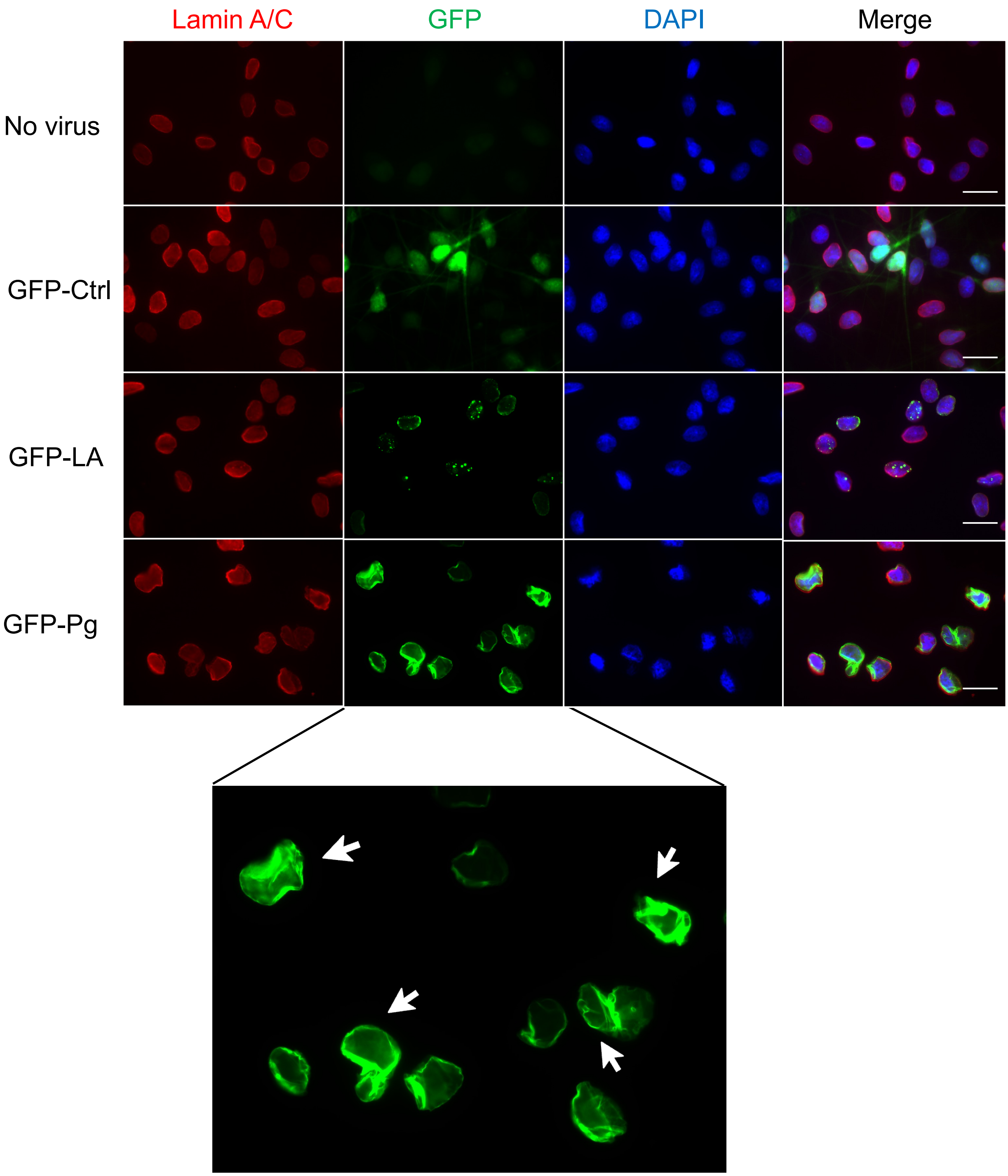

Figure S5.

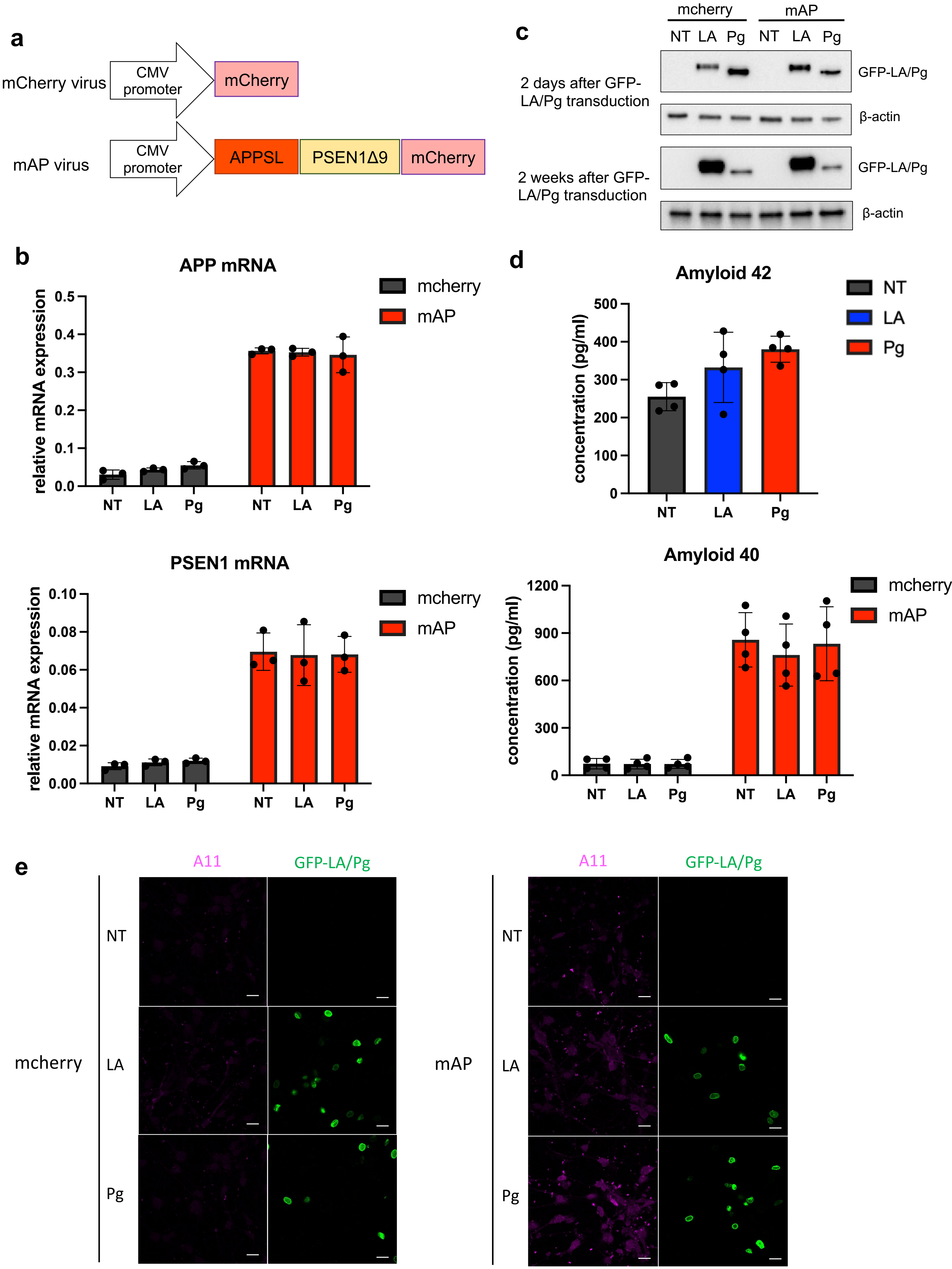
a
mCherry virus
CMV promoter
mCherry
mAP virus
CMV promoter
APPSL
PSEN1Δ9
mCherry
c
mcherry
mAP
NT LA Pg NT LA Pg
2 days after GFP-LA/Pg transduction
GFP-LA/Pg
β-actin
2 weeks after GFP-LA/Pg transduction
GFP-LA/Pg
β-actin
b
APP mRNA
relative mRNA expression
mcherry
mAP
NT LA Pg NT LA Pg
PSEN1 mRNA
relative mRNA expression
mcherry
mAP
NT LA Pg NT LA Pg
d
Amyloid 42
concentration (pg/ml)
NT
LA
Pg
Amyloid 40
concentration (pg/ml)
mcherry
mAP
NT LA Pg NT LA Pg
e
A11
GFP-LA/Pg
mcherry
mAP
NT
LA
Pg

Figure S6.

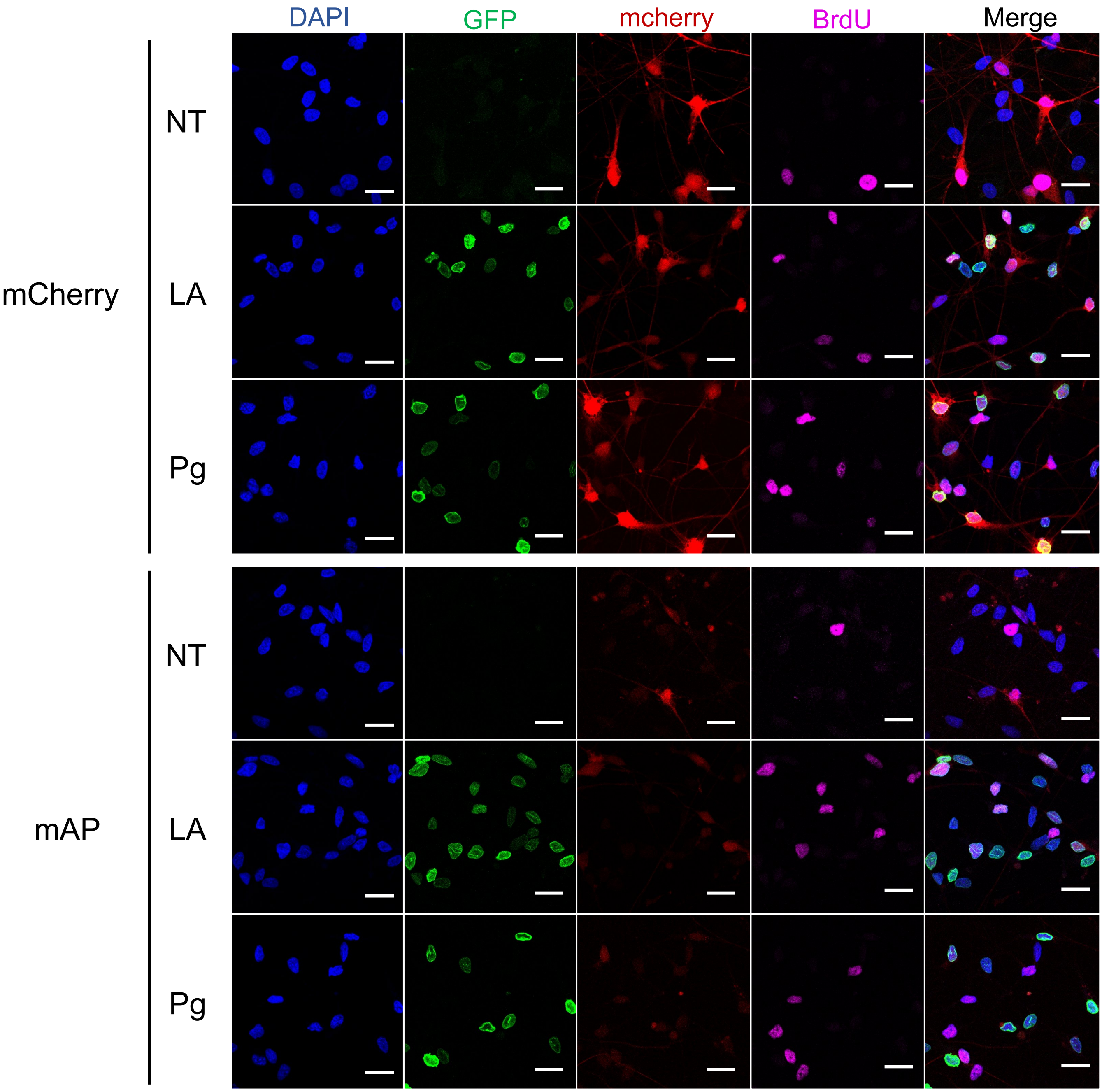

Figure S7.

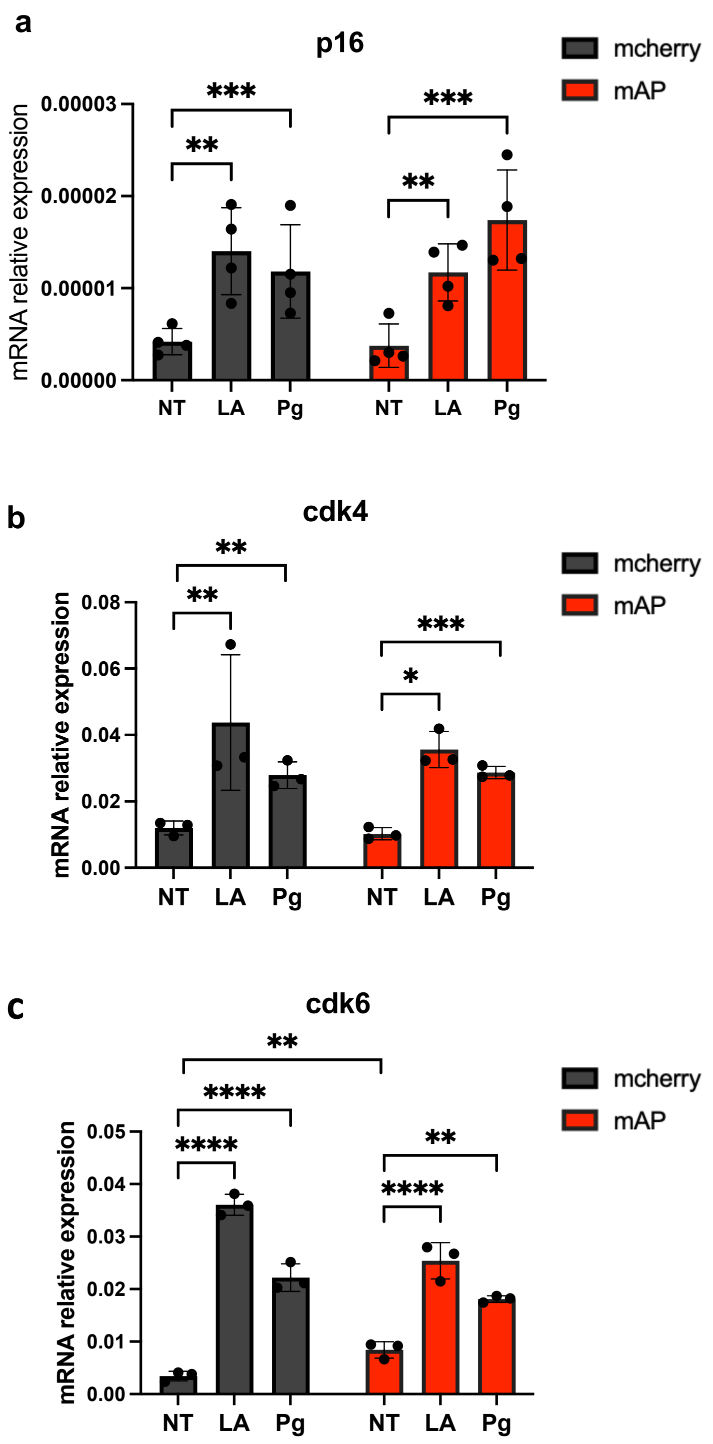
a
p16
mcherry
mAP
mRNA relative expression
0.00003
0.00002
0.00001
0.00000
***
**
***
**
NT
LA
Pg
NT
LA
Pg
b
cdk4
mcherry
mAP
mRNA relative expression
0.08
0.06
0.04
0.02
0.00
**
**
***
*
NT
LA
Pg
NT
LA
Pg
c
cdk6
mcherry
mAP
mRNA relative expression
0.05
0.04
0.03
0.02
0.01
0.00
**
****
****
**
****
NT
LA
Pg
NT
LA
Pg

Figure S8.

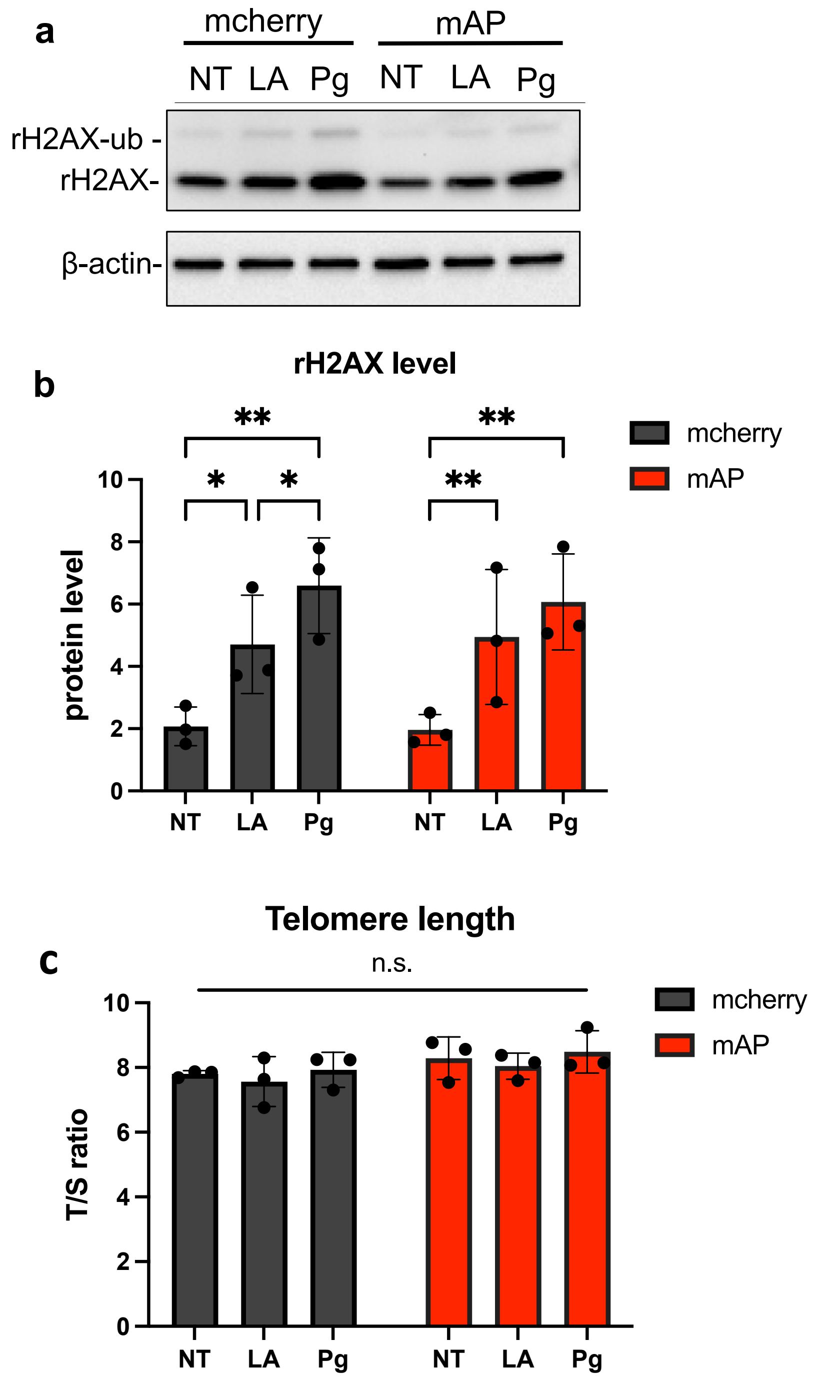
a
mcherry
mAP
NT LA Pg NT LA Pg
rH2AX-ub -
rH2AX-
β-actin-
b
rH2AX level
**
*
*
**
**
mcherry
mAP
protein level
0
2
4
6
8
10
NT LA Pg NT LA Pg
c
Telomere length
n.s.
mcherry
mAP
T/S ratio
0
2
4
6
8
10
NT LA Pg NT LA Pg

Figure S9.

Gene	Forward (5'-3')	Reverse (5'-3')
**ACTB**	CTGGAACGGTGAAGGTGACA	AAGGGACTTCCTGTAACAATGCA
**Lamin A**	GCAACAAGTCCAATGAGGACCA	CATGATGCTGCAGTTCTGGGGGCTCTGGAT
**Lamin C**	CTCAGTGACTGTGGTTGAGGA	AGTGCAGGCTCGGCCTC
**Lamin B1**	GGAGAATCGTTGTCAGAGCCTT	TGCGGCTTTCCATCAGTTCT
**APP**	TGGGTTCAAACAAAGGTGCA	GTTCTGCTGCATCTTGGACA
**PSEN1**	GCAGTATCCTCGCTGGTGAAGA	CAGGCTATGGTTGTGTTCCAGTC
**P16**	GAGCAGCATGGAGCCTTC	CGTAACTATTCGGTGCGTTG
**Cdk4**	CCATCAGCACAGTTCGTGAGGT	TCAGTTCGGGATGTGGCACAGA
**Cdk6**	CCAGGCAGGCTTTTCATTCA	AAGTATGGGTGAGACAGGGC
**Telomere**	CGGTTTGTTTGGGTTTGGGTTTGGGTTTGGGTTTGGGTT	GGCTTGCCTTACCCTTACCCTTACCCTTACCCTTACCCT
**β-globin**	GCTTCTGACACAACTGTGTTCACTAGC	CACCAACTTCATCCACGTTCACC

Figure S9. qPCR and telomere length assay primer list
